# Staphylococcal Toxic Shock Syndrome Toxin-1 Induces the Translocation and Secretion of High Mobility Group-1 Protein from Both Activated T Cells and Monocytes

**DOI:** 10.1155/2008/512196

**Published:** 2008-11-04

**Authors:** Shirin Kalyan, Anthony W. Chow

**Affiliations:** ^1^Vancouver Hospital & Health Sciences, Diamond Health Care Centre, Department of Medicine, Vancouver, BC, Canada V5Z 1M9; ^2^Division of Infectious Diseases, Department of Medicine, University of British Columbia, Vancouver, BC, Canada V5Z 3J5

## Abstract

High mobility group box-1 (HMGB-1) is a DNA-binding protein secreted by activated monocytes and has been identified as a key late mediator of endotoxic shock. We investigated the regulation of HMGB-1 in human peripheral blood mononuclear cells (PBMCs) following stimulation with the staphylococcal superantigen, toxic shock syndrome toxin-1 (TSST-1), and found that TSST-1, like LPS, induced the secretion of HMGB-1 from human PBMC. However, unlike monocyte-driven sepsis caused by endotoxin, translocation and secretion of HMGB-1 mediated by TSST-1 was dependent on the presence of both activated T cells and monocytes. Furthermore, we show that nuclear HMGB-1 is released from TSST-1 stimulated T cells. This finding presents a basis for investigating the potential of targeting HMGB-1 for the treatment of toxic shock syndrome, and provides further insight on the role of HMGB-1 in the cross-talk between activated monocytes and T cells.

## 1. INTRODUCTION

High mobility group box-1 (HMGB-1) protein is a 30 kDa nonhistone nuclear DNA binding protein that is shown to have an extracellular role in inflammation, cell differentiation, adherence, and motility [1–3]. Extracellular HMGB-1 was initially identified as amphoterin, a heparin-binding protein promoting neurite outgrowth in the perinatal rat brain [4]. HMGB-1 is now regarded as an endogenous danger signal that is passively released by necrotic cells or actively secreted by stimulated cells [5]. Wang et al. discovered that this pivotal protein was a late mediator of endotoxin shock [6, 7]. Activated macrophages and monocytes secrete this inflammatory molecule by a process requiring acetylation of the protein, which permits its translocation from the nucleus to secretory lysosomes [8]. Unlike TNF*α* and IFN*γ* that appear within the first 4–6 hours post-LPS treatment in mice, HMGB-1 serum levels rise between 16 and 32 hours after LPS administration. Furthermore, neutralizing antibodies directed at HMGB-1 rescued mice from lethal endotoxemia even when administered 24 hours after sepsis initiation [7, 9]. For these reasons, HMGB-1 is viewed as an attractive therapeutic target for various inflammatory disorders including endotoxic shock [10].

Toxic shock syndrome toxin-1 (TSST-1) is a superantigen (sAg) secreted by some strains of *S. aureus,* and this sAg was subsequently identified as the major causative agent of toxic shock syndrome [11]. In contrast to LPS-induced sepsis, which is primarily mediated by stimulated monocytes, toxic shock syndrome induced by sAg's requires the cross-linking of V*β*-specific regions of the *α* *β* T cell receptor (TCR) to class II major histocompatibility complex (MHC II) molecules on antigen presenting cells (APCs) [12]. In addition to massive T cell proliferation, this trimolecular interaction leads to the uncontrolled release of various proinflammatory cytokines [13], which are pivotal to the pathogenesis of TSS. In the present study, we sought to determine whether HMGB-1 also plays a central role in TSST-1-induced hyperinflammatory responses. We found that TSST-1 mediates the translocation and subsequent secretion of intracellular HMGB-1 from resting human PBMC. Unlike previous studies that found that monocytes, but not T cells, released HMGB-1 upon stimulation with either LPS or TNF*α* [6, 7, 14], we found that the loss and subsequent secretion of intracellular HMGB-1 induced by TSST-1 was dependent on the cooperative interaction of both T cells and monocytes, and both cell types mobilized HMGB-1 upon TSST-1 treatment.

## 2. MATERIALS AND METHODS

### 2.1. Toxin purification

Recombinant TSST-1 was purified from culture supernatants of *S. aureus* strain RN4220 previously transformed to carry the *tst* gene, using both preparative isoelectric focusing and chromatofocusing [15]. Toxin purity was assessed by silver staining after sodium dodecyl sulfate-polyacrylamide gel electrophoresis on 14% acrylamide gels, and LPS activity was undetectable by the *Limulus* amoebocyte lysate gelation (sensitivity limit, 10 pg/mL).

### 2.2. Preparation of cells and culture conditions

Fresh human PBMCs from healthy donors were obtained by Ficoll-Paque PLUS (Amersham Biosciences Corp., Piscataway, NJ, USA) density centrifugation, and cultured in 96-well U-bottom plates at 1.5 × 10^6^ cells/mL in complete culture medium consisting of RPMI 1640 (StemCell Technologies Inc., Vancouver, BC, Canada), 10% heat-inactivated fetal bovine serum (HyClone Laboratories Inc., Logan, Utah, USA), 2 mM L-glutamine (StemCell), 25 mM Hepes buffer (StemCell), and 2 ug/mL of polymyxin B sulphate (Sigma-Aldrich Corp., St. Louis, Mo, USA). For analysis of secreted HMGB-1 in cell culture supernatants, PBMCs were plated in 24-well flat-bottom plates in Opti-MEM I reduced serum medium (Gibco). THP-1 cells (human monocytic cell line) were obtained from ATCC and kept in recommended growth media until use, at which time the cells were plated at a concentration of 1.5 × 10^6^ cells in Opti-Mem I medium. T cell depletion of human PBMC was performed using a column-free method of magnetic bead separation (EasyCep from StemCell Technologies) by positive selection of T cells using an anti-CD3 conjugated antibody according to manufacturer's directions. This procedure left the monocytes negatively selected for and untouched for the purpose of further experimental investigations.

### 2.3. Treatment of PBMC with TSST-1 or LPS

Purified PBMCs were plated in either 24-well or 96-well culture plates overnight at 37°C in 5% CO_2_ prior to stimulation with 1 nM TSST-1 or 500 ng/mL LPS (Sigma), where noted. Culture supernatants were collected at 24 hours post-TSST-1 treatment, microcentrifuged at 800xg for 5 minutes, and frozen at –70°C until protein analysis.

### 2.4. Detection of HMGB-1 by fluorescent microscopy

PBMCs were first surface-stained for expression of CD3 with mouse antihuman CD3 IgG (Pharmingen BD Biosciences, Mississauga, ON, Canada) followed by antimouse IgG antibody conjugated to Alexa594 (Molecular Probes); cells were subsequently fixed using Cytofix buffer (Pharmingen). For the intracellular detection of HMGB-1, cells were permeabilized with Cytoperm (Pharmingen) and incubated for 30 minutes (4°C) with rabbit polyclonal anti-HMGB-1 antibodies (Orbigen BioCarta, San Diego, Calif, USA) diluted in blocking buffer (phosphate buffered saline, 3% FBS) followed by a secondary antibody with Alexa488-conjugated goat antirabbit IgG. The anti-HMGB-1 antibodies were raised against the peptide sequence corresponding to amino acids 166–181 that were previously shown to be specific for HMGB-1 and not HMGB-2 [16]. Surface expression of HMGB-1 on PBMC was established by culturing cells (37°C, 5% CO_2_) directly on coverslips to allow attachment of adherent cells overnight before stimulation with 1 nM of TSST-1. Cells were then fixed and surface-stained as above (but without the permeabilization step). Fluorescent-labeled PBMCs were subsequently stained with Hoechst 3342 nuclear dye (molecular probes), and mounted on slides using prolong antifade reagent (molecular probes). Cells were visualized with an AxioPlan II fluorescence microscope equipped with a CCD camera using Northern Eclipse software (Epix) for acquisition of images. Images were taken with the 63x oil immersion objective lens, and Adobe Photoshop 6.0 software was used for image layout.

### 2.5. Flow cytometric analysis of surface-expressed HMGB-1 on differentiated cells

Surface-expressed HMGB-1 was analyzed by flow cytometry (FACSCalibur Flow Cytometry System, BD BioSciences Pharmingen) using the anti-HMGB-1 antibody and secondary Alexa488-conjugated antibody described above in conjunction with phycoerythrin (PE)-conjugated anti-CD3 and anti-CD14 antibodies (BD BioSciences Pharmingen), with a minimum of 10 000 events collected for each sample.

### 2.6. Western blot analysis of secreted HMGB-1

PBMC or THP-1 culture supernatants were concentrated 10-fold from original volume using Amicon Ultra centrifugal filters with a molecular weight cut-off of 10 kDa (Millipore). Some culture supernatants had been further prepared using the SDS-PAGE Clean-Up Kit (Amersham) according to the manufacturer's directions prior to running on a 12% polyacrylamide gel. Western blotting was performed by semidry transfer of proteins (Trans-Blot SD Semi-Dry Electrophoretic Transfer Cell, BioRad) onto an Immobilon-P PVDF membrane (Millipore) which had been blocked for 1 hour at room temperature with 1% BSA, 0.5% Tween in Tris buffered saline (TBS) prior to overnight incubation (at 4°C) with rabbit polyclonal anti-HMGB-1 antibody. The membrane was subsequently incubated with antirabbit IgG horse-radish-peroxidase (HRP)-conjugated secondary antibody for 1 hour at room temperature on a shaker. Detection of HMGB-1 was performed using super signal substrate (Pierce) and developed as well as analyzed using the Alpha Innotech 3400 Gel Documentation system (Alpha Innotech, Calif, USA).

### 2.7. Statistical analysis

 Statistical analysis was performed using Prism 3.0 software package (GraphPad). The proportion of PBMCs expressing HMGB-1 following treatment with TSST-1 or RPMI in different donors was compared by paired Student's *t*-test. Differences were considered significant if *P* < .05.

## 3. RESULTS AND DISCUSSION

### 3.1. Secretion of cell-surface expressed HMGB-1 from differentiated and adherent cells 24 hours post-TSST-1 stimulation

**Figure 1:**
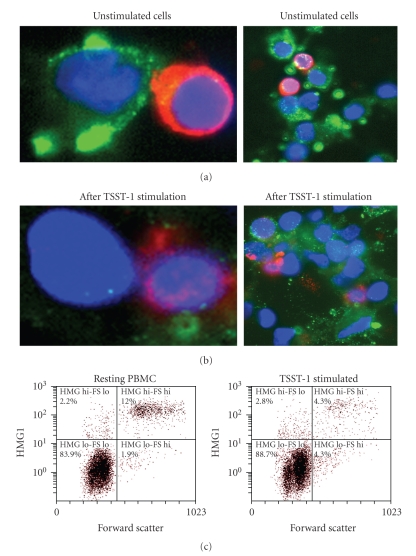
*Secretion of membrane-associated HMGB-1 in the extracellular milieu following TSST-1 treatment.* Extracellular expression of HMGB-1 on adherent PBMC in complete growth medium (a) or after TSST-1 treatment. (b) Anti-HMGB-1: green fluorescence (Alexa488); nucleus: blue (Hoechst 3342 nuclear dye); anti-CD3: red (Alexa594). Close-up views from representative fields are depicted in the left panels. (c) Flow cytometry analysis of surface-expressed HMGB-1 in PBMC 24 hours following TSST-1 stimulation. Left panel, resting or PBMC treated; right panel, 24 hours after TSST-1 stimulation. Surface HMGB-1 expression significantly decreased after TSST-1 stimulation (12.0% versus 4.3% in the donor shown; 7.3 ± 3.69% prior to stimulation versus 3.3 ± 1.32% post-TSST-1 treatment from 4 different donors; *P* < .05, paired Student's *t*-test).

**Figure 2:**
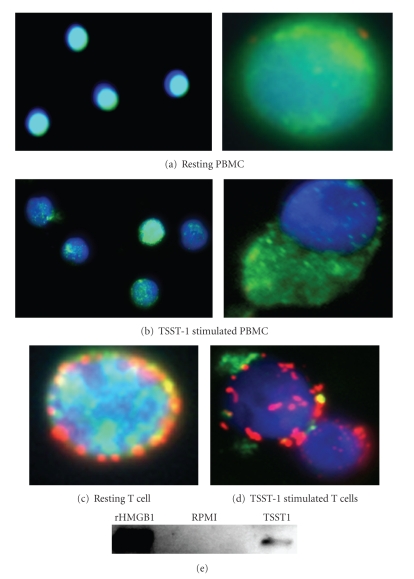
*Nuclear translocation of HMGB-1 in human PBMC following TSST-1 stimulation.* Human PBMCs were treated with either 1 nM TSST-1 or RPMI medium for 10 hours, and the intracellular expression of HMGB-1 was detected by confocal fluorescent microscopy. Close-up views from representative fields are depicted in the right panels. (a) Resting (RPMI) PBMC, intracellular HMGB-1 (green) was seen to be localized primarily in the nucleus (blue). (b) At 10 hours following stimulation with 1 nM TSST-1, HMGB-1 was seen to translocate into the extracellular space. (c) Fluorescent microscopy image of resting T cells, and (d) TSST-1 stimulated T cells in the process of actively translocating intracellular HMGB-1 10 hours following toxin treatment. T cells were detected by staining with antihuman CD3 antibodies conjugated to Alexa594 (red). (e) Detection of HMGB-1 by immunoblot in the culture supernatant of human PBMC 24 hours after treatment with 1 nM TSST-1 or RPMI medium.

Some differentiated as well as adherent cells express HMGB-1 on the cell surface, and this cell membrane-associated HMGB-1 has been referred to as amphoterin to distinguish it from intracellular HMGB-1 [17]. Extracellular amphoterin expression has been well described previously, and has been investigated using various methods including subcellular fractionation, immunogold electron microscopy, and mRNA localization studies [18]. We examined the surface expression of HMGB-1 (amphoterin) in human PBMC after culturing cells over a 48-hour period in 96-well U-bottom plates in complete growth medium. After this incubation time, we identified a population of differentiated cells that expressed high levels of HMGB-1 in the absence of any further treatment with exogenous stimulants (Figure 1(a)). Because they are cells in suspension, freshly purified PBMCs do not express amphoterin (i.e., cell-surface expressed HMGB-1). Expression of cell-surface-associated HMGB-1 took place over the 48-hour time course that PBMCs were cultured, and cells of monocyte lineage had become adherent. T cells, which were identified by costaining with anti-CD3 and Alexa594-conjugated antimouse IgG (red), did not express amphoterin since they remain in suspension, and the surface expression of HMGB-1 appears to be a property of adherent cells; however, all cells express intracellular HMGB-1 (as shown in Figure 2). Our observations corroborate with those of Rouhiainen et al. [18], who reported the accumulation of amphoterin in the extracellular space of cells bearing process extensions, and HMGB-1 surface expression was inhibited in cells without these processes. Following TSST-1 stimulation, there was a significant loss of amphoterin from the cell surface from the population of cultured adherent cells that had surface expression of HMGB-1, as observed by fluorescent microscopy (Figures 1(a) and 1(b)), and quantified by flow cytometric analysis (Figure 1(c)). Flow cytometry also confirmed that the high-level surface expression of amphoterin was confined to a subpopulation of PBMC having a high forward scatter corresponding to the monocyte/macrophage population (estimated to be between 3% and 12% of resting PBMC among the donors tested). More than half of these amphoterin-expressing cells released HMGB-1 upon TSST-1 stimulation (7.3 ± 3.69% prior to stimulation versus 3.3 ± 1.32% post- TSST-1 treatment; *P* < .05, paired Student's *t*-test from 4 different donors). Flow cytometric analysis, to our knowledge, has not been utilized previously for quantifying the surface expression of HMGB-1, and we found it to be a useful tool to evaluate the change in extracellular HMGB-1 expression.

### 3.2. Translocation and secretion of intracellular HMGB-1 from human PBMCs 10 hours post-TSST-1 stimulation

To investigate the potential change in intracellular HMGB-1 expression upon TSST-1 exposure, human PBMCs were treated with 1 nM of TSST-1 for 10 hours, and then subsequently evaluated for the translocation of intracellular HMGB-1 by fluorescence microscopy (Figure 2). As reported previously [19], HMGB-1 in resting PBMC is expected to be localized primarily in the nucleus (Figure 2(a)). However, 10 hours after treatment with 1 nM TSST-1, a significant proportion of PBMCs had decreased their intracellular stores of HMGB-1 (Figure 2(b)). Previous studies that used either LPS or the inflammatory cytokines IFN*γ* or TNF*α* [6, 7] as stimulants found that only macrophages, but not T cells, translocated and subsequently secreted nuclear HMGB-1. However, we found that T lymphocytes in human PBMCs also underwent a change in intracellular HMGB-1 expression following TSST-1 treatment (Figures 1(c) and 1(d)).

To verify that HMGB-1 was actively secreted from TSST-1 treated PBMCs, we performed a Western blot analysis of cell culture supernatants collected 24 hours post-TSST-1 treatment (Figure 2(e)).

### 3.3. Requirement of activated T cells in TSST-1-induced nuclear translocation and secretion of HMGB-1 in human PBMC

**Figure 3:**
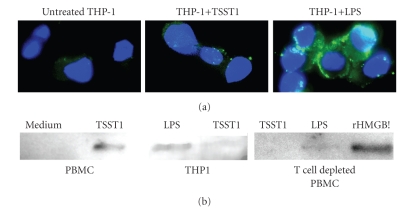
*Requirement of T cells for HMGB-1 secretion following TSST-stimulation.* (a) THP-1 cells or T cell-depleted human PBMCs were stimulated with either TSST-1 (1 nM), LPS (500 ng/mL), or RPMI medium for 24 hours, and surface expression of HMGB-1 was examined by fluorescence microscopy. (b) The secretion of HMGB-1 in supernatants of THP-1 cells (left panel) or T cell-depleted PBMC (right panel) 24 hours following treatment with LPS, TSST-1, or RPMI medium control was examined by immunoblot. rHMGB-1 (15 ng) was used as a positive control.

Undifferentiated and nonadherent monocytes in suspension do not express cell-surface-associated HMGB-1. However, during the first steps in differentiation, monocytes actively transport this molecule to their cell surface, and HMGB-1 is subsequently secreted into the extracellular milieu upon further stimulation [17]. To further investigate the requirement of T cells for nuclear translocation and secretion of HMGB-1 following TSST-1 stimulation, we treated the human monocytic cell line, THP-1, with either TSST-1 (1 nM), LPS (500 ng/mL), or leaving them untreated in RPMI medium. THP-1 cells express basal levels of both MHC Class II required for TSST-1 activation and CD14 required for LPS-induced activation [20]. As shown in Figure 3, TSST-1 treatment failed to induce surface expression of HMGB-1 on undifferentiated THP-1 cells, in contrast to stimulation with LPS. We confirmed this observation in primary cells using human PBMCs that were markedly depleted of their T cell pool that was stimulated with either TSST-1 or LPS (Figure 3(a)). Western blot analysis also verified that THP-1 cells and T cell-depleted PBMC secreted detectable amounts of HMGB-1 upon LPS stimulation, but not TSST-1 treatment (Figure 3(b)). Collectively, these results confirm our previous studies which established that both monocytes and T cells are required for the secretion of TNF*α* and IL-1*β* from human PBMC stimulated with highly purified TSST-1 [13].

## 4. CONCLUSION

Various cell types have recently been identified as contributing to the extracellular pool of HMGB-1, including human umbilical vein endothelial cells [21], platelets [18], pituicytes, and macrophages [3]. Our finding that HMGB-1 is secreted by T cells following TSST-1 stimulation may parallel the observation made by Semino et al. [22] who found that distinct NK cell subsets secreted HMGB-1 which served to differentiate autologous dendritic cells, and this event was modulated by environmental stimuli. HMGB-1 was shown to induce monocytes to differentiate into dendritic cells that specifically polarized T cells to give a Th1 response [19], a feature characteristic of the effect of TSST-1 stimulation on human PBMC. We previously demonstrated that the cytokine secretion and costimulatory molecule expression by human PBMC following TSST-1 stimulation follows a bimodal pattern [23], with the first phase peaking at ~3 hours poststimulation, and a second burst at ~24 hours poststimulation. In light of the secretion of HMGB-1 into the extracellular milieu at this later time point, it will be worthwhile to determine whether the second inflammatory burst and the upregulation of costimulatory molecules, such as CD86, CD40, as well as HLA-DR (which peaked at 48 hours) [23], are in direct response to the secretion of HMGB-1 that is considered the late mediator of sepsis [24].

The best studied ligand for extracellular HMGB-1 is RAGE (receptor of advanced glycation end products) which is upregulated on activated macrophages as well as endothelial cells [25]. Previous studies have aimed to determine how superantigen-activated T cells adhere to vascular endothelial cells and induce vascular injury [26]. It was also shown that rHMGB-1 elicited proinflammatory responses on endothelial cells [25]. It would, therefore, be of interest to further investigate the role of HMGB-1 and RAGE in the inflammatory response and vascular endothelial injury mediated by TSST-1. It is anticipated that the model of TSST-1-induced inflammation, which necessitates the bridging of V*β*2-specific T cells and MHC class II molecules bearing antigen presenting cells, will provide a useful means to study the role of HMGB-1 in T cell-monocyte interactions, Th1 polarization, and the ensuing immune response leading to tissue injury. Finally, this study also provides a rationale to investigate the potential to target HMGB-1 for therapy for inflammatory disorders induced by TSST-1, such as toxic shock syndrome, as it is currently being investigated for gram negative sepsis [9]. It should be noted that the inflammatory response to different staphylococcal exotoxins varies [27], and it would be of clinical interest to investigate the role and regulation of HMGB-1 in the context of other superantigens and its potential as a primary target for superantigen-mediated diseases.
